# QTL mapping of a Brazilian bioethanol strain links the cell wall protein-encoding gene GAS1 to low pH tolerance in *S. cerevisiae*

**DOI:** 10.1186/s13068-021-02079-6

**Published:** 2021-12-16

**Authors:** Alessandro L. V. Coradini, Fellipe da Silveira Bezerra de Mello, Monique Furlan, Carla Maneira, Marcelo F. Carazzolle, Gonçalo Amarante Guimaraes Pereira, Gleidson Silva Teixeira

**Affiliations:** 1 Department of Genetics, Evolution, Microbiology, and Immunology, Institute of Biology, University of Campinas, Rua Monteiro Lobato 255, Campinas, 13083-862 Brazil; 2 Molecular and Computational Biology Section, Department of Biological Sciences, University of Southern California, Los Angeles, CA 90089-2910 USA

## Abstract

**Background:**

*Saccharomyces cerevisiae* is largely applied in many biotechnological processes, from traditional food and beverage industries to modern biofuel and biochemicals factories. During the fermentation process, yeast cells are usually challenged in different harsh conditions, which often impact productivity. Regarding bioethanol production, cell exposure to acidic environments is related to productivity loss on both first- and second-generation ethanol. In this scenario, indigenous strains traditionally used in fermentation stand out as a source of complex genetic architecture, mainly due to their highly robust background—including low pH tolerance.

**Results:**

In this work, we pioneer the use of QTL mapping to uncover the genetic basis that confers to the industrial strain Pedra-2 (PE-2) acidic tolerance during growth at low pH. First, we developed a fluorescence-based high-throughput approach to collect a large number of haploid cells using flow cytometry. Then, we were able to apply a bulk segregant analysis to solve the genetic basis of low pH resistance in PE-2, which uncovered a region in chromosome X as the major QTL associated with the evaluated phenotype. A reciprocal hemizygosity analysis revealed the allele *GAS1*, encoding a β-1,3-glucanosyltransferase, as the casual variant in this region. The *GAS1* sequence alignment of distinct *S. cerevisiae* strains pointed out a non-synonymous mutation (A631G) prevalence in wild-type isolates, which is absent in laboratory strains. We further showcase that *GAS1* allele swap between PE-2 and a low pH-susceptible strain can improve cell viability on the latter of up to 12% after a sulfuric acid wash process.

**Conclusion:**

This work revealed *GAS1* as one of the main causative genes associated with tolerance to growth at low pH in PE-2. We also showcase how *GAS1*^*PE-2*^ can improve acid resistance of a susceptible strain, suggesting that these findings can be a powerful foundation for the development of more robust and acid-tolerant strains. Our results collectively show the importance of tailored industrial isolated strains in discovering the genetic architecture of relevant traits and its implications over productivity.

**Supplementary Information:**

The online version contains supplementary material available at 10.1186/s13068-021-02079-6.

## Background

*Saccharomyces cerevisiae* strains that are resistant to acidic environments are desirable in many relevant biotechnological processes, from probiotics, food and beverage industries [1–3], to bioethanol production [4, 5]. Usually, bioethanol is produced from a fermentation process driven by *S. cerevisiae* in which carbon sources from raw feedstock, such as corn, beet, wheat, and sugarcane are employed [6]. The alcohol production is based on the fermentation of available 6-carbon sugars from juice and/or starch (first-generation ethanol, E1G), or 5 and 6-carbon sugars present in lignocellulosic material and made available through hydrolysis (second-generation ethanol, E2G) [7]. In the latter, acidic fermentation environments arise as a consequence of the acid treatment of lignocellulosic material, which produces high quantities of acetic acid and other inhibitory by-products, such as 5-hydroxymethylfurfural and furfural [8]. The combination of low pH and inhibitory by-products perturb the intracellular pH homeostasis, inducing cell death and consequent loss of productivity [9, 10]. Therefore, an additional step of pH neutralization is often required before proceeding to the fermentation process increasing the operational costs [11].

In Brazil, the second-largest bioethanol producer in the world, distilleries commonly use a Melle-Boinot fermentation operation [12], in which high-density cell cultures are applied and the yeast recycled by centrifugation and acid washing to initiate a subsequent fermentation. In short, yeast cells are exposed to an acid treatment with dilute sulfuric acid (H_2_SO_4_) for up to 2 h before being reintroduced into a new vessel for a subsequent cycle of fermentation [13]. This unit operation aims to reduce bacterial contamination and prepare the cells for a new fermentation batch, reducing the need for yeast propagation, thus generating a more productive and less consuming process [14]. However, the severity of the process decreases the viability of the yeast population and consequently reduces productivity [15]. Also, this process may work as a cell bottleneck, limiting the variety of strains that can be used in the process [16].

Given the harsh conditions faced by yeasts in the Brazilian industrial fermentation process, indigenous strains have been praised for their robust background that allows higher ethanol productivity while facing burdening stress factors. In this scenario, *S. cerevisiae* strain Pedra-2 (PE-2) has been described as efficient industrial yeast, able to outperform native yeasts and dominate the fermentation process within a few cycles of fermentation and recycling [5, 17, 18]. The molecular analysis of JAY270 (PE-2 industrial isolate) shed light on its highly heterozygous genome architecture, which harbors structural polymorphisms between homologous chromosomes, especially in peripheral regions. The extreme heterozygosity and plasticity of the PE-2 genome and transcriptome have been hypothesized to contribute to its rapid adaptability to the industrial fermentative environment [19]. In fact, analysis of PE-2 performance during growth at harsh condition demonstrated its superior phenotype when exposed to low pH [17, 20], high temperature and oxidative stress conditions [19].

Responses to acid stress in *S. cerevisiae* have been thoroughly revised elsewhere [21]. Besides the effects on cell viability [22] and fermentation productivity [23], low pH can induce oxidative stress [24] and enhance ethanol toxicity [25].

Acid stress can be triggered by the presence of week organic acids (WOA) such as lactic, acetic and formic acid or by inorganic acids mainly sulphuric and hydrochloric. The presence of WOA at external pH below the weak acid pK_a_ value, facilitate the entrance of the undissociated form of the acid (RCOOH) on the cell by simple diffusion [1]. Once in the neutral cytosol, the chemical dissociation of the weak acid occurs leading to the release of protons (H^+^) that strongly decrease the pH of cell cytoplasm [26]. Thus, the main cell response to this internal acidification involves increasing vacuolar H^+^-ATPase (V-ATPase) activity [27–34]. Due to the high energetic cost of proton cell extrusion, additional mechanisms to prevent cytosolic acidification are necessary which include alteration of the molecular composition and physical properties of plasma membrane and cell wall [1]. In this scenario, genes related to cell wall remodeling and synthesis of cell wall major polysaccharides—chitin and β-glucan, and polymers of mannose—are transcriptionally responsive to weak acids [29, 35–39]. Stiffness of the cell wall caused by an increased content of cell wall β-glucans was recently associated to yeast resistance to acetic acid [40] demonstrating that cell wall remodeling may also play an important role on cell response to acid stress by WOA.

The capacity to maintain and remodel cell wall also appears as the main physiological response to yeast adaptation to inorganic acid exposure. When an inorganic acid is present in the environment, a high concentration of protons is generated. However, distinct from WOA, protons diffuse poorly across the plasma membrane and so the cytoplasm pH is not drastically reduced [41]. Thus, cell exposure to inorganic acids mainly affects cell wall structure and organization [4, 41, 42]. Previous studies on yeast transcriptional response to low pH demonstrated that adaptation of yeast involves mechanisms that include induction of cell wall integrity (CWI) genes and general stress response (GSR) pathway mainly due to the action of protein kinase C (PKC) [36, 42–45]. In particular, genes related to transport, protein anchoring and synthesis of the β-1,3-glucan chain on yeast cell wall are up-regulated after cell treatment with sulphuric acid [42, 44]. Together these results point to the possibility of the cell wall remodeling, in special changes in its polysaccharides content, as a general response of the cell to acidic environment. Therefore, changes in cell wall structure and content induced by exposure to inorganic acids may also lead to an improved resistance to growth in an acidic environment in the presence of either inorganic or organic acids.

In this study, we performed a Quantitative Trait Loci (QTL) mapping approach to unravel the genetic architecture behind the high acid resistance phenotype of the PE-2 strain. We first hypothesized that currently exposure of PE-2 to repetitive cycles of acid treatment with dilute sulfuric acid (H_2_SO_4_) in Brazilian bioethanol mills conferred to PE-2 an increased tolerance to low-pH environments. This hypothesis was confirmed by the superior performance of PE-2 over other 41 Saccharomyces sp. strains during growth at low pH (2.5). Using a high-throughput approach to en masse phenotype of segregants at pH 2.1 followed by a bulk segregant analysis approach (BSA), allows the identification of 2 major *loci* on chromosome X and XIII. Furthermore, Reciprocal hemizygosity analysis (RHA) revealed the *GAS1* allele from PE-2 as the main causal variant associated to loci on Chromosome X. Sequence alignment of different *S. cerevisiae GAS1* alleles pointed out a persistent non-synonymous mutation within industrial and wild-type isolates, in contrast to laboratory strains. Finally, we showcased that low pH resistance phenotype can be recovered in susceptible strains with the insertion of PE-2 mutated *GAS1*. So far, this study presents itself as the first to explore the genetic basis of tolerance to growth at low pH in *S. cerevisiae* using a QTL mapping approach. Also, this work may help to better understand the molecular mechanisms underlying relevant industrial traits and, consequently, could foment the development of more robust strains for different applications.

## Results

### Screening of parent strains for genetic mapping

**Fig. 1:**
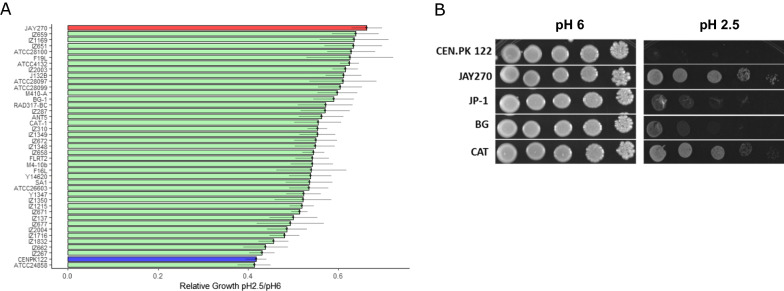
Phenotyping of yeast strains in low pH. **A** Relative growth of 41 industrial, laboratory and wild-type *Saccharomyces sp.* strains in low pH, represented by the ratio of the colony size at pH 2.5 and neutral (pH 6). The relative growth of JAY270 (PE-2 derived) and CEN.PK122 strains were highlighted in red and blue, respectively. **B** Spot test of CEN.PK122, JAY270 and other Brazilian bioethanol industrial strains (JP-1, BG and CAT) at pH 2.5 and control (pH 6)

The growth performance of the PE-2 isolate, JAY270 (MATa/MATα), and other 40 *Saccharomyces sp.*—including laboratory, industrial and wild-type isolates—was assessed by measuring their relative colony size during growth on rich media at low (2.5) and neutral (6) pH (Fig. 1A). The JAY270 strain displayed the best growth performance in comparison to the other evaluated strains, presenting only 33% colony size reduction at low pH. On the other hand, the laboratory strain CEN.PK112 [46] ranked amongst the strains with the lowest performance. JAY270 also outperforms other typically used bioethanol strains (Fig. 1B). Therefore, considering tolerance to growth at low pH, JAY270 and CEN.PK112 were selected as the superior and inferior strain, respectively.

**Fig. 2:**
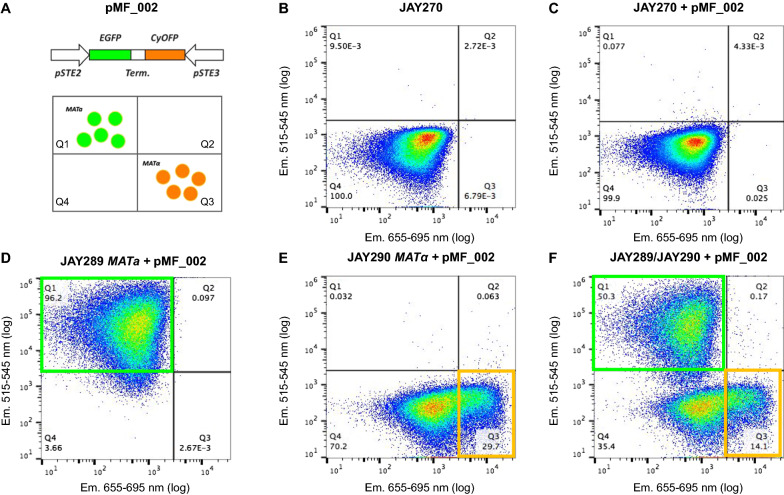
Patterns of fluorescence emission in strains transformed with pMF_002 vector and analyzed by flow cytometric. **A** Schematic representation of the pMF_002 vector and simplification of expected results. **B** Fluorescence pattern of the diploid strain JAY270 without pMF_002 vector. **C** Fluorescence pattern of the diploid strain JAY270 containing the vector pMF_002. **D** Fluorescence pattern of the haploid strain JAY289 (MATa) transformed with vector pMF_002. **E** Fluorescence pattern of the haploid JAY290 (MATα) transformed with pMF_002. **F** Fermentation pattern of a heterogeneous population comprising both haploids JAY289 (MATa) and JAY290 (MATα) transformed with vector pMF_002. Specific MATa cells emitting green fluorescence are expected to form a population on quadrant 1 (Q1) while specific MATα cells emitting orange fluorescence are expected to fall on quadrant 3 (Q3)

JAY270 segregant haploids were collected using fluorescence-activated cell sorting (FACS) by flow cytometry through a distinct mating-type approach. For this, we constructed vector pMF_002, comprising the reporter gene enhanced green fluorescent protein (EGFP) fused to the MATa-specific STE2 (STErile 2) promoter, and cyan orange fluorescent protein (CyOFP1), to MATα-specific STE3 (STErile 3) promoter (see “Methods” for details). When using only a fluorescein isothiocyanate (FITC) filter, EGFP and CyOFP1 are excited in distinct wavelengths of 515/545 (green) and 655/695 (orange), respectively. Thus, when expressing pMF_002, MATa cells emit green fluorescence and MATα cells orange fluorescence, while diploid cells (MATa/MATα) and tetrads do not display any fluorescence. This technique allowed the separation and distinct collection of 1084 stable JAY270 haploid cells (Fig. 2). A similar strategy has been previously published by Treusch et al. [47], and here adapted to be suitable for more commonly available excitation wavelengths.

Before proceeding to the phenotyping of the isolated segregants, a minimum inhibitory concentration (MIC) was established for acid tolerance—i.e. the minimum pH value capable of totally inhibiting the growth of at least one haploid cell. Therefore, the colony size of 48 randomly selected JAY270 segregants was evaluated in decreasing values of pH (Additional file 1: Fig. S1), and a pH of 2.1 was selected as the MIC. Next, the panel of 1,084 JAY270 collected haploids was tested for their growth at pH 2.1 to evaluate their phenotypic response.

**Fig. 3:**
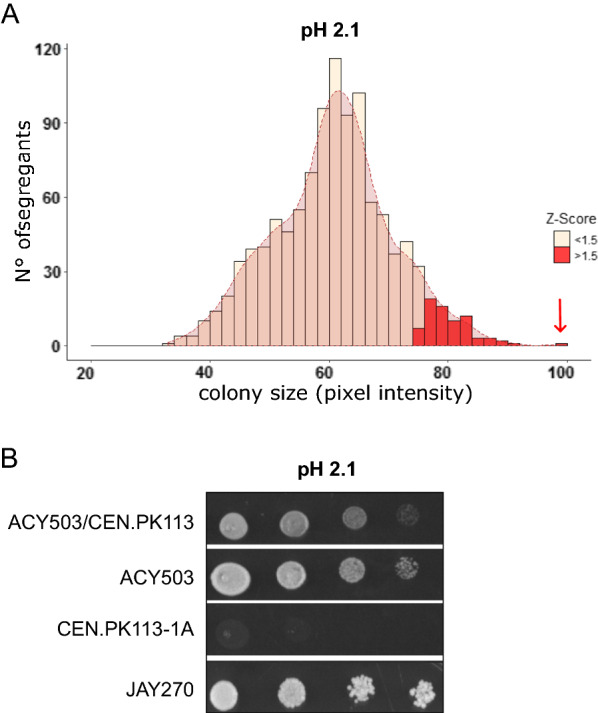
Phenotypic analysis of JAY270 segregant haploids in low pH. **A** Distribution histogram of the colony size of 1,084 collected JAY270 haploids at pH 2.1. The darker red quadrant represents the haploids with the best tolerance to low pH, with Z-score > 1.5 and the red arrow point to the growth performance of the superior segregant ACY503. **B** Spot assay to evaluate the growth performance of the hybrid ACY503/CEN.PK113-1A (MATa/MATɑ), haploids ACY503 (MATa) and CEN.PK113-1A (MATɑ) and the parental diploid JAY270 at low pH 2.1

The normal distribution of the segregants’ colony size in the evaluated condition reveals the quantitative characteristic of the low pH tolerance phenotype in the JAY270 strain (Fig. 3A). In this population, haploid ACY_503 (MATa) presented the largest colony size at pH 2.1, therefore was selected as the superior parental strain. Strain CEN.PK113-1A (MATα), CEN.PK122 segregant that presented reduced growth at low pH, was chosen as the inferior parental strain. The crossing between the selected strains resulted in the diploid ACY_503/CEN.PK113-1A. The hybrid diploid and both parental strains were submitted to a colony spot assay to confirm their phenotypes in an acid medium (Fig. 3B). As expected, the resistance and susceptibility of parental haploid strains ACY_503 and CEN.PK 113-1A, respectively, was confirmed. In addition, it is important to notice that the resulting hybrid ACY_503/CEN.PK113-1A presented low pH resistance similar to the superior parental ACY_503, indicating that the genetic architecture that underlies the acid tolerance in JAY270 should be dominant.

### Selection of the two pools of segregants with extreme phenotypes

**Fig. 4:**
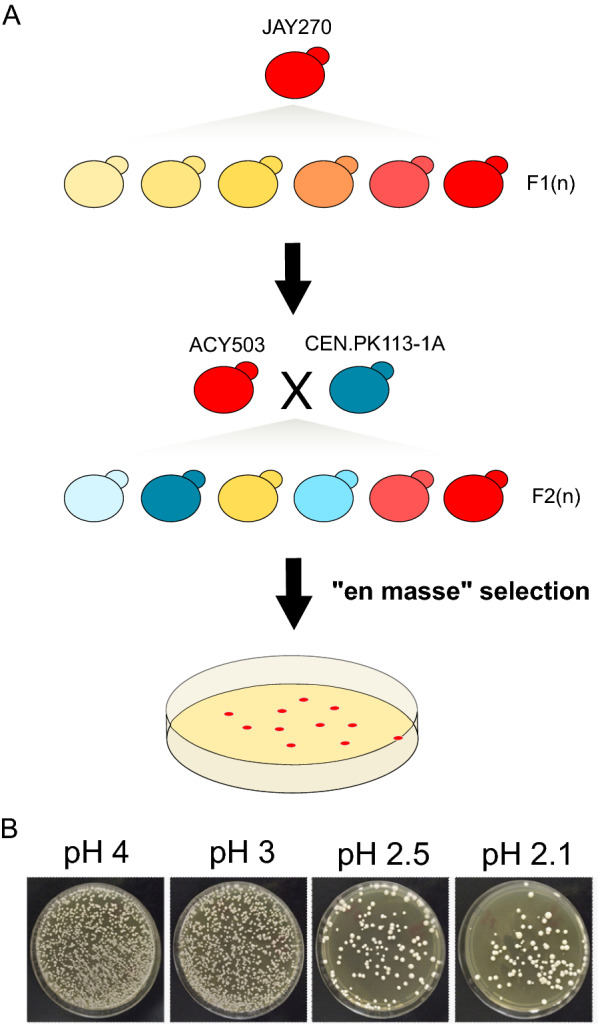
En masse selection strategy to obtain a pool of low-pH-tolerant segregants from the ACY503/CEN.PK113-1A cross. **A** Schematic view of an en masse collection of a pool of ACY503/CEN.PK113-1A acid-tolerant segregants. For the QTL mapping, initially a highly low-pH-resistant JAY270 segregant was selected (ACY503) and further crossed with a haploid of the opposite phenotype. The hybrid transformed with pMF_002 was sporulated and segregants were further collected using a cell sorter coupled with a flow cytometer in growing challenges of pH resistance. **B** Image of YPD plates with decreasing values of pH (4, 3, 2.5 and 2.1) where 500.000 ACY503/CEN.PK113-1A segregants were collected in flow cytometry. 79 segregants obtained at pH 2.1 were considered a “*high resistance pool*” and used for QTL mapping

The use of the plasmid pMF_002 allowed the application of the bulk segregant analysis (BSA) approach to map potential QTL related to tolerance to growth at low pH on PE-2. Therefore, ACY_503/CEN.PK113-1A was transformed with the vector pMF_002, sporulated and its segregants were collected using a cell sorter coupled to a flow cytometer. In order to select segregants in the positive extreme of low pH tolerance phenotypic distribution, the isolated F_2_ population was collected on YPD plates containing decreasing values of pH: 4, 3, 2.5, and 2.1 (Fig. 4). In this scenario, after 96 h of incubation, 79 segregants—*petite* colonies were excluded from this examination—were obtained and labeled as the “*high resistance pool*” (Additional file 2: Fig. S2A).

One disadvantage of BSA is that it does not allow for the selection of a pool of segregants with inferior phenotype (i.e. haploids susceptible to low pH), since en masse selection is provided by increasing the restrictive growth condition. Thus, we randomly selected 500 haploids that were further phenotyped using a colony spot assay, resulting in 79 segregants with reduced growth at pH 2.1, that were labeled as the *“low resistance pool”* (Additional file 2: Fig. S2B).

### Identification of QTL related to growth tolerance at low pH of the JAY270 strain

Genomic DNA of four samples: (i) PE-2-derived superior haploid ACY_503; (ii) laboratory susceptible haploid CEN.PK113-1A; (iii) 79 segregants from the *high resistance pool*; and (iv) 79 segregants from the *low resistance pool*—were subjected to whole-genome sequencing analysis using the Illumina HiSeq 4000 platform that generated millions of 2 × 100 paired-end reads and genome coverages of 213, 170, 267, and 210x, respectively. The reads from the two parental strains and the two pools were first aligned to the CEN.PK113-7D reference genome sequence [48] to identify single nucleotide polymorphisms (SNPs). A total of 47,659 highly credible SNPs between high and low resistance pools were selected for QTL analysis.

**Fig. 5:**
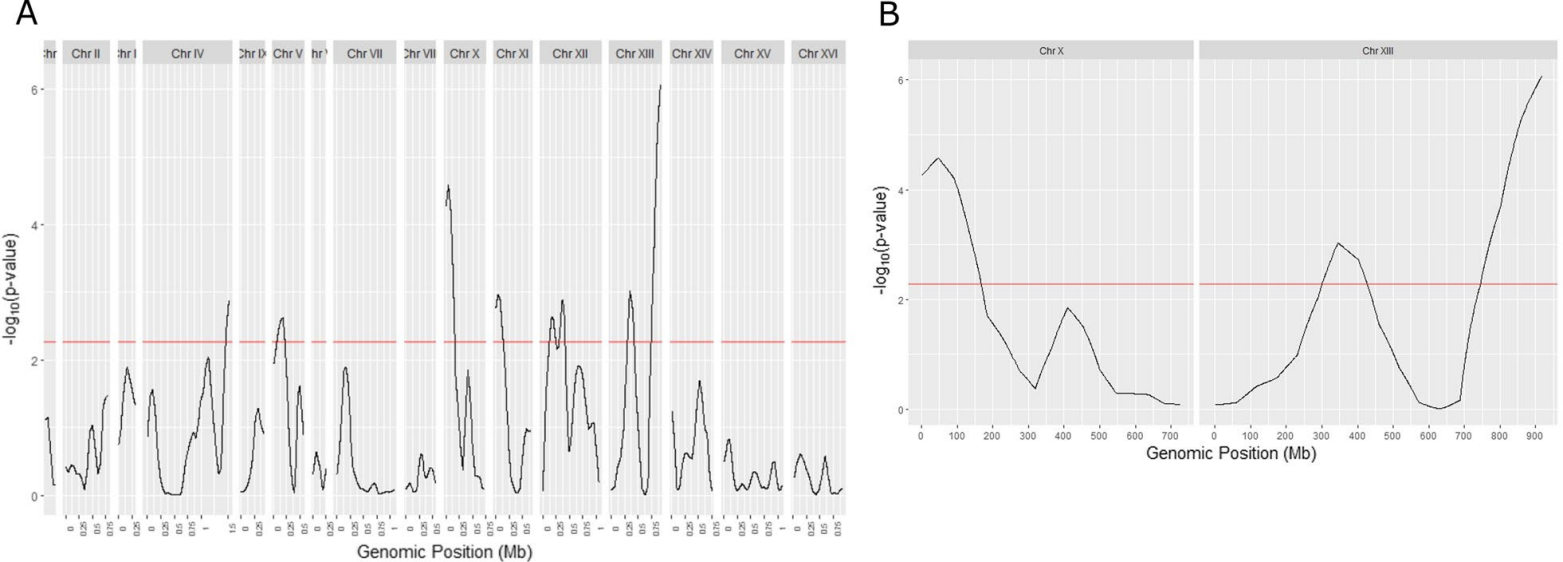
Mapping of the *loci* related to low pH tolerance by pooled-segregant whole-genome sequencing analysis. The X-axis indicates the chromosome’s position; Y-axis indicates the value of – log10(p-value) calculated for windows of 80 kb. The *p*-values are calculated for each SNP and are estimated from the null distribution of G’, which assumes no QTL. The threshold value for – log10(p-value) is indicated by the red line. SNPs that showed p-values above the FDR are considered enriched, deviating from the null distribution hypothesis. **A** QTL mapping of the whole genome. **B** QTL mapping of chromosomes X and XIII that presented − log_10_(p-value) above the threshold of 2.3

The G’ values for each SNP were calculated using an 80-kb sliding window, and the p-value graph was plotted to identify the candidate peaks (Fig. 5). Peak regions of − log_10_(p-value) above the threshold of 2.3 were defined as candidate QTL regions comprising alleles responsible for the evaluated phenotype. The mapping shows two major peaks: at chromosome X and XIII with − log_10_ (p-value) of 6.12 and 4.7, respectively. Because chromosome XIII presented the highest value of − log_10_(p-value), therefore representing a region enriched with SNPs more statistically relevant in the *high resistance pool*, it was selected as the major QTL and used in further analysis.

The mutations present within a region of 50 kb surrounding the highest peak position on chromosome XIII (897,054 bp) were analyzed and annotated. Initially, all SNPs located in non-coding regions and that confer synonymous mutations were excluded from the list of potential candidates. Further, we used the genome sequence of 14 *S. cerevisiae* strains, available at Saccharomyces Genome Database (SGD) [49], to infer the non-synonymous coding SNP frequency in this population (Table 1). Since extreme low pH tolerance is not a common trait in yeast a low frequency of the candidate SNP was expected. Mutations with frequencies lower than 30% were, therefore, classified as potential candidates. Finally, we also considered the attributed function for each gene in which the filtered SNP is located to narrow down the number of potential causative alleles.

**Table 1:** Non-synonymous mutations present in genes within a 50-kb window in chromosome XIII peak (897,054 bp) of low-pH phenotype QTL mapping

	YME2					UBP15			GAS1	NIP1
	255	817	1270	1408	2037	916	2410	2589	631	1388
S288C	A	G	A	G	G	C	A	A	A	T
CEN.PK	G	T	T	A	T	T	T	T	A	T
ACY503	A	G	A	G	G	C	A	A	G	A
X2180-1A	A	G	A	G	G	C	A	A	A	T
SEY6210	A	G	A	G	G	C	A	A	A	T
W303	A	G	A	G	G	C	A	A	A	A
JK9-3d	A	G	A	G	G	C	A	A	A	T
FL100	A	G	A	G	G	T	T	T	A	A
D273-10B	A	G	A	G	G	C	A	A	A	T
Sigma1278b	A	G	A	G	G	C	A	A	A	T
RM11-1a	G	T	T	A	T	C	A	A	G	A
SK1	G	T	T	A	T	C	T	A	G	A
Y55	G	T	T	A	T	C	A	A	G	A
BY4741	A	G	A	G	G	C	A	A	A	T
BY4742	A	G	A	G	G	C	A	A	A	T

	YMR310C		GLC8	ELP6	TGL3	DIA1		FET4	
	1776	442	605	685	4	1495	707	950	1656
S288C	T	T	C	C	G	T	T	C	T
CEN.PK	T	T	C	C	G	T	T	C	T
ACY503	C	C	G	A	A	C	C	T	C
X2180-1A	T	T	C	C	G	T	T	C	T
SEY6210	C	T	C	C	G	T	T	C	T
W303	T	C	G	A	A	C	C	C	T
JK9-3d	C	T	C	C	G	T	T	C	T
FL100	T	C	G	A	A	C	C	C	T
D273-10B	T	T	C	C	G	T	T	C	T
Sigma1278b	T	T	C	C	G	T	T	C	T
RM11-1a	T	C	C	C	G	C	C	C	T
SK1	C	C	G	A	A	C	C	C	T
Y55	T	C	C	C	A	C	C	C	T
BY4741	T	T	C	C	G	T	T	C	T
BY4742	T	T	C	C	G	T	T	C	T

Using this approach, we were able to identify 4 potential candidate genes on chromosome XIII related to low pH resistance: *GAS1*, *ELP6*, *GLC8,* and *FET4*. A description of the function of each gene and the non-synonymous uncommon mutations present in ACY_503 can be found in Table 2.

**Table 2:** Possible causative alleles related to low-pH-resistance phenotype at chromosome XIII with uncommon non-synonymous coding mutations

Gene	Function^a^	Mutations in ACY503^b^
*GAS1*	Beta-1,3-glucanosyltransferase; required for cell wall assembly and also has a role in transcriptional silencing; genetic interactions with histone H3 lysine acetyltransferases GCN5 and SAS3 indicate previously unsuspected functions for Gas1 in DNA damage response and cell cycle regulation	A631G
*ELP6*	Elp6p is part of the six-subunit elongator complex, which is a major histone acetyltransferase component of the RNA polymerase II holoenzyme responsible for transcriptional elongation	C685A
*GLC8*	Regulatory subunit of protein phosphatase 1 (Glc7p); involved in glycogen metabolism and chromosome segregation; proposed to regulate Glc7p activity via conformational alteration; protein abundance increases in response to DNA replication stress	C605G
*FET4*	Although originally identified as a low-affinity iron(II) permease, Fet4p has since been shown to import several other transition metal ions, including copper and zinc	C950T; T1656C

^a^Available at https://www.yeastgenome.org/

^b^Non-synonymous uncommon mutations

### Validation of the causative genes within chromosome XIII QTL

**Fig. 6:**
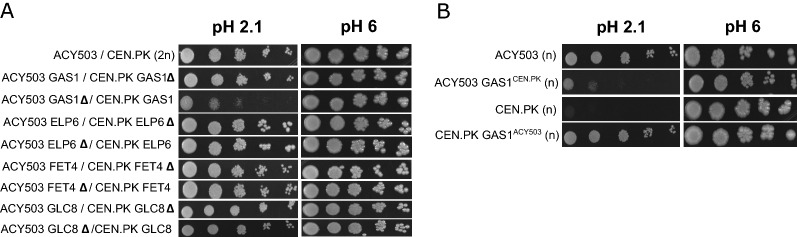
Identification of the causative gene *GAS1* on chromosome XIII by using reciprocal hemizygosity analysis of candidate alleles of low pH resistance phenotype. **A** Spot growth assay of the hemizygous diploids for *GAS1*, *ELP6*, *GLC8,* and *FET4*. The reciprocal hemizygote containing the *GAS1* allele from ACY503 outperformed the one presenting the allele of CEN.PK113-1A(CEN.PK) at pH 2.1. Spot growth assay on control condition (pH 6) was also performed to check if hemizygous deletion did not affect strain fitness. **B** Spot assay on low pH of ACY503 and CEN.PK113-1A (CEN.PK) strains with interchanged *GAS1* allele. The swap of *GAS1* allele between the strains remarkably improved CEN.PK113-1A tolerance to low pH, while the opposite occurred with ACY503 expressing *GAS1* allele from CEN.PK113-1A

The validation of the candidate alleles was performed through RHA. Four pairs of hemizygous ACY_503/CEN.PK113-1A strains were generated, in which each pair retained a single copy of the superior ACY_503 or inferior CEN.PK113-1A parental alleles—*GAS1*, ELP6, *GLC8* or FET4 (Fig. 6A). The hemizygous strains for each candidate allele were tested for growth at pH 2.1 and the phenotypic response assessed through colony spot assays. The results showed that phenotypic variance is present on *GAS1* hemizygotes, and that the strain expressing the *GAS1*^ACY_503^ allele presented superior growth compared to the one that harbors *GAS1*^CEN.PK113-1A^. On the other hand, the response of the other hemizygous diploid strains for the remaining 3 genes not changed on the evaluated condition, compared to the wild-type hybrid.

Next, we sought to identify if swapping *GAS1* allele between susceptible and tolerant parental would induce different responses in their acid tolerance phenotype (Fig. 6B). Interchanging the *GAS1* allele between both strains significantly improves CEN.PK113-1A tolerance to low pH, while it reduces ACY_503’s, corroborating with the results obtained by RHA and strongly suggesting that the *GAS1* allele contributes to low pH tolerance in segregant ACY_503, and consequently in PE-2 strains.

Compared to the CEN.PK113-1A allele, the *GAS1* allele of ACY_503 contains one non-synonymous point mutation, within its coding sequence (position 887,003 bp on chromosome XIII). This mutation accounts for a nucleotide exchange at position 631 from adenine to guanine and results in a threonine to alanine substitution. The *GAS1* allele from ACY_503 also harbors a synonymous mutation at position 1314 bp (i.e*.* a cytosine replacing thymine).

Next, we compared GAS1 sequence from ACY_503 and other 1053 *Saccharomyces cerevisiae* strains containing laboratorial and wild isolate whose genome sequencing is available at Saccharomyces genome database (SGD) [49] and Peter et al. [50]. The frequency of non-synonymous mutation found on ACY_503 is high (96.8%) in the total *S. cerevisiae* analyzed population (Table 3). Interestingly, this mutation seems common throughout the majority of wild-type isolated strains, including all the Brazilian bioethanol isolates, while it is not found on laboratory ones (Additional file 3: Table S1). The same analysis was carried out for the synonymous mutation at nucleotide 1314 and distinct from the non-synonymous one it presents a low frequency (5.1%) on total analyzed population. However, this frequency increases to 76.9% when the analyzed population includes only isolates from Brazilian Bioethanol process (Table 3).

**Table 3:** Evaluation of GAS1 SNPs frequency in 1053 distinct *Saccharomyces cerevisiae* strains

SNP location in GAS1	Nucleotide	Total *Saccharomyces cerevisiae* population		Brazilian bioethanol strain population	
		Number of strains	SNP frequency (%)	Number of strains	SNP frequency (%)
631	A (reference)	32	3.0	0	0
	G	1019	96.8	39	100
	R (A or G)	2	0.2	0	0
1314	T (reference)	54	92.0	7	17.9
	C	969	5.1	30	76.9
	Y (C or T)	30	2.8	2	5.1

### Analysis of the CEN.PK113-1A *GAS1*^ACY_503^ mutant tolerance to acid-wash treatment with H_2_SO_4_ solution.

As previously stated, the E1G production process in Brazilian mills has the peculiarity of using the Melle-Boinot operation, characterized by fed-batch fermentation cycles with high-density cell recycle. Yeast cells are recycled up to 3 times per day and, between each recycling step, face an acid-wash treatment with sulfuric acid (pH ~ 1.5) that aims to reduce bacterial contamination and flocculation [16, 51]. Thus, after linking the *GAS1* allele to PE-2 tolerance to growth at low pH, we sought to explore if it is possible to increase tolerance to an acid-wash treatment in a naturally susceptible strain after allele swap (i.e. by constructing a strain expressing *GAS1*^ACY503^ allele).

**Fig. 7:**
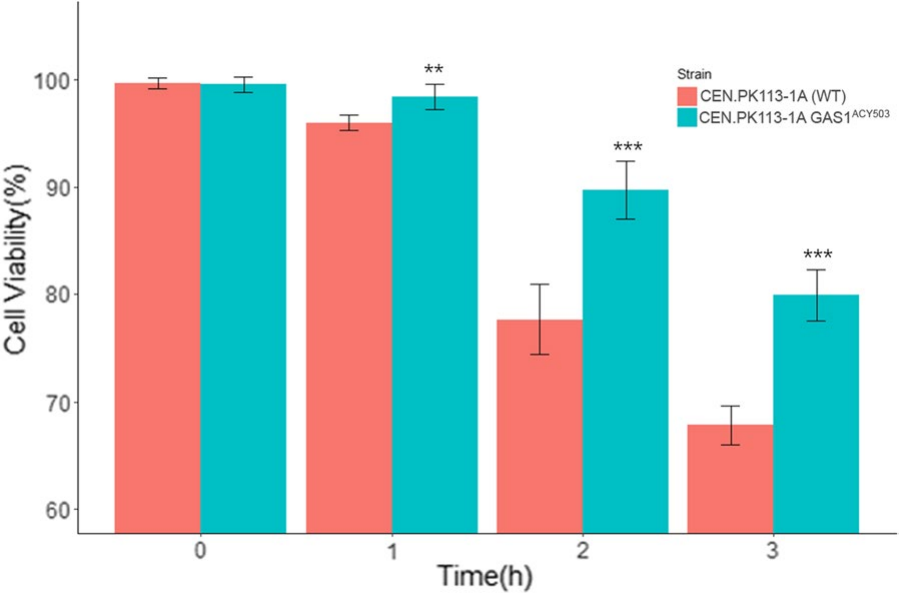
Cell viability after acid-wash assay. Cell viability of CEN.PK113-1A and its mutant containing the *GAS1* allele from the tolerant strain ACY503 was assessed each hour during 3 h of treatment with H_2_SO_4_ solution

For this purpose, wild-type CEN.PK113-1A strain and its mutant CEN.PK113-1A *GAS1*^ACY_503^ were submitted to an acid treatment in which cells were exposed to a diluted H_2_SO_4_ solution with pH 1.5 for up to 3 h. Viability was assessed every hour, revealing that the CEN.PK113-1A strain containing the mutated *GAS1* allele presents superior cell viability when compared to the wild type. After 3 h of exposure to H_2_SO_4_ solution, CEN.PK113-1A cell containing GAS1 allele maintaining 12% more viable cells when compared to wild type CEN.PK113-1A (Fig. 7).

## Discussion

QTL mapping approaches have been extensively used to unravel the genetic basis of complex traits in a wide range of organisms [52]. In the yeast *S. cerevisiae*, QTL mapping approaches have facilitated the association between genetic variants and industrially relevant traits [53]. In this scenario, wild-type isolated strains present a higher degree of genetic and natural selection-driven diversity compared with domesticated laboratory ones, which facilitate their use to resolve the genetic basis of so-desired relevant traits. In this study, a bioethanol industrial strain—PE-2, isolated from Brazilian mills—was used to unravel potential genetic variants associated with tolerance to growth at low pH in *S. cerevisiae*.

An initial evaluation of growth performance of JAY270 (PE-2 derivate) and other 40 *S. cerevisiae* strains cultivated at a low pH condition confirmed the superior phenotype of PE-2, especially when compared to the laboratory strain CEN.PK122. In fact, PE-2 persistence on the ethanol production process in Brazilian mills has already been associated with its resistance to the acid-wash cell recycle step typically performed on Brazilian E1G production process [5, 20]. On the other hand, common laboratory strains such as CEN.PK122 are typically cultivated under standard conditions that include slightly acidic pH and consequently do not undergo natural selection for this specific condition. Our initial result corroborates with the idea that the harsh conditions faced by yeast strains during bioethanol production produce tailored strains that can easily outcompete non-adapted ones. Thus, industrial isolated strains from the Brazilian bioethanol process, such as PE-2, SA-1, CAT-1, BG-1 can be a good source of genetic variability to explore the genetic basis of industrial relevant traits.

In order to investigate the genetic architecture of PE-2 acid tolerance, we first developed a high-throughput fluorescence-based approach to isolate a large number of yeast segregants. In comparison to other BSA approaches, such as X-QTL [54], our method allows the rapid generation of large mapping populations without extensive strain engineering. Despite the similarity to the fluorescence-based approach described by Treusch et al. [47], our method has the advantage of decreasing the number of wavelength gates necessary for segregant isolation. It makes use of the eGFP and CyOFP1 fluorescent proteins, which are excited at wavelengths of 515/545 (green) and 655/695 (orange), respectively, via a single FITC filter. This is the first report of the use of CyOFP1 in a fluorescent-based approach for yeast cell separation.

The developed fluorescence-based approach was successfully applied to isolate a large pool of segregants for BSA-based QTL mapping. BSA is an efficient approach for detection of major QTLs associated with complex traits in yeast [55–63]. This approach relies on phenotyping a progeny from a cross and genotyping two subsets of these offspring presenting opposite phenotypes [64]. The developed fluorescence-based approach was successfully applied to isolate a large pool of segregants (500.000) from a cross between the ACY_503 (PE-2 derivative) and CEN.PK113-1A strains. The segregants were collected in crescent challenging conditions of low pH, which allowed the selection of a pool of 79 superior and inferior haploids—representing less than 0.01% of the total analyzed population.

By analyzing differential SNPs presence in both pools (ΔSNP-index), we were able to identify a major QTL located at the end of chromosome XIII. Although the QTL region encompasses a genomic window of approximately 150 kb containing several genes, we focused our analysis on the 50 kb window surrounding the detected QTL peak. We also applied a protein function analysis as well as non-synonymous mutation information in these genes, narrowing down the number of potential candidates to only 4. Through generation of engineered reciprocal hemizygotes, we were able to identify *GAS1* as the causative allele on the major QTL at chromosome XIII. Furthermore, by interchanging the *GAS1* alleles between ACY_503 and CEN.PK113-1A, we remarkably increased CEN.PK113-1A tolerance to low pH condition. Both results indicate *GAS1* as the causal variant on QTL at chromosome XIII responsible for low pH tolerance on PE-2 strain.

*GAS1* encodes a cell wall-bound 1,3-beta-glucanosyltransferase involved in the formation and maintenance of 1,3-beta-glucan, which is the major polysaccharide of the cell wall (see review on [65]). The Gas1p is a GPI-anchored glycoprotein of 125–130 kDa localized at the plasma membrane and is a member of the GH72 family of β-1,3-glucanosyltransferases that also include the *Candida albicans* pH-responsive proteins—CaPhr1p and CaPhr2p [66]. The enzyme is characterized by an N-terminal catalytic domain of about 310 residues (D23–P332), known as the β-(1,3)-glucan transferase domain (GluTD), a cysteine-rich region containing a motif of eight cysteines (C370–C462) and a serine-rich region in which 28 serines are clustered in a region between residues S485 and S525 [67].

A comparison between ACY_503 and CEN.PK113-1A alleles showed the presence of two distinct mutations at nucleotides positions 631 and 1314, non-synonymous and synonymous, respectively. Thus, the non-synonymous mutation at A211 amino acid residue is located at N-terminal catalytic domain and more precisely on α-helices domain between the two activity glutamates residues E161 and E262 necessary for *GAS1* activity as b-(1,3)-glucanase and b-(1,3)-glucanosyltransferase [68].

A more comprehensive analysis of the presence of both SNPs in other *S. cerevisiae* strains revealed that the non-synonymous mutation is common on wild strains isolated from distinct sources—e.g. wineries, bioethanol industries and oak trees. On the other hand, this mutation is absent in laboratory domesticated strains such as S288C, CEN.PK and W303. Yeasts are known to be organisms with the capacity to survive and ferment in a more acidic environment—pH 4–5 [41, 69]. Acidification of the extracellular environment can be a consequence of natural processes occurring during fermentation, presence of competing microorganisms producing organic acids or as a consequence of human interference during a biotechnological process [4]. However, acidic environments are rare in controlled laboratory conditions, where yeast growth is typically carried out in standard conditions that include neutral and controlled pH. The lack of selective pressure in laboratory growth conditions may have contributed to the loss of beneficial genetic variants associated with low pH resistance on laboratory strains.

Regarding the synonymous mutation at nucleotide 1314, it appears enriched in the Brazilian bioethanol strain population when compared to the total analyzed population (Table 3). This result may point to a positive selection of this mutation on the strains used in Brazilian bioethanol process. Although synonymous mutations are not expected to cause phenotypic changes, there is emerging evidence that it may affect cell fitness by altering translational efficiency, mRNA stability and also perturbing co-translational protein folding mechanisms [70–72].

The Gas1p role on yeast low pH resistance may be related to activation of cell wall integrity (CWI) pathway. The CWI pathway is responsible for maintenance and function of the yeast cell wall and its mechanism is controlled by the regulatory cascade led by protein kinase [73]. In summary, the stress sensor Mid2 mediates a response to acidic conditions that leads to activation of the Rlm1 transcription factor through phosphorylation of MAP kinase Slt2p/Mpk1p [74, 75]. G*as1*Δ mutant strains show hypersensitivity to low pH and present higher levels of dually phosphorylated Slt2, which may help explain the connection between Gas1p and CWI pathway. Some studies have also demonstrated the existence of a synthetic interaction between Gas1p, Slt2p and Rlm1 [76]. Collectively these results point that maintenance of cell wall structure is an important response to low pH stress. Corroborating with this idea, transcriptomic analysis of yeast cells growing under low pH showed that GAS1 and other genes related to cell wall biogenesis appear up-regulated as a response to the damages caused by strong inorganic acids such as sulphuric acid [42], a stress response that may be triggered by CWI pathway activation.

Recently, Ribeiro et al. [40] demonstrated that Gas1p also play a role in yeast cell wall response to stress caused by organic acid such as acetic acid. The presence of 60 mM of acetic acid (pH 4.0) in the medium up-regulates the transcription of β-1,3-glucanosyltransferase encoded by gene *GAS1* that leads to an increased content of cell wall β-glucans. This correlation between the increased levels of *GAS1*’s transcripts and the content of glucan in the cell wall suggests that at least partially, the cell wall remodeling under acetic acid presence is due to the action of β-1,3-glucanosyltransferase encoded by *GAS1*. This remodeling is essential for preventing acetate (dissociated form of acetic acid due to the low pH) entry through passive diffusion into the cell.

The *GAS1* was also identified as responsible for low pH resistance of the multiple-stress-tolerant yeast *Issatchenkia orientalis* (*Pichia kudriavzevii*) [68]. Matsushika et al. have screened on *S. cerevisiae* a genomic DNA library of *I. orientalis* identifying *loGAS1* as the allele responsible for low pH resistance and also demonstrating that expression of lo*GAS1* in *S. cerevisiae* (S288C) improved its ethanol fermentation ability at pH = 2. In a complementary study, the same group demonstrated that *S. cerevisiae GAS1* (*ScGAS1*) expression is pH-dependent and increases in low pH conditions [77]. Also, overexpression of *ScGAS1* improved growth and ethanol production under acid stress conditions, although the stress tolerance was inferior to that of the *IoGAS1*-overexpressing strain. The DNA sequences of both genes—*loGAS1* and *ScGAS1*, possess approximately 60% of similarity [68]. Interestingly, by analyzing and comparing the amino acids profile from loGas1p with the Gas1p produced by the ACY_503 and CEN.PK113-1A alleles, we found that the non-synonymous mutation present in the ACY_503 allele result in the same amino acid (alanine) as at that position in the *loGAS1* gene.

Finally, to build evidence that *GAS1*^ACY_503^ may also play a role to PE-2 tolerance to acid-wash treatment of the cell recycle process on Brazilian bioethanol mills, we analyzed the cell survival rate of the susceptible strain CEN.PK113-1A and its mutant CEN.PK113-1A *GAS1*^ACY_503^ when submitted to a H_2_SO_4_ solution. The result showed that the strain harboring mutant *GAS1* allele preserves up to 12% more viable cells after 2 and 3 h of acid treatment. This result might be indicative of PE-2 strain tolerance to the acid-wash treatment and its prevalence on fermentation vessels, as described by Basso et al. [16]. Furthermore, this result also opens the possibility of using genetic engineering to develop more robust strains for ethanol production (E1G and E2G) and also other biotechnological processes where cells experienced loss of cell viability or productivity due to the acidic environment.

## Conclusion

In this study, we explored the industrial isolated strain PE-2 resistance to growth at low pH. By using a high-throughput approach, we were able to isolate and collect thousands of segregants and apply a BSA to map the QTL related to this phenotype. Following, a RHA approach allowed us to uncover the allele *GAS1* as one of the causal variant related to low pH tolerance by PE-2. Further, we used reverse genetic engineering to improve tolerance to acidic pH of the strain CEN.PK113-1A, demonstrating that *GAS1*^ACY_503^ is able to confer this phenotype. This study is, up to date, the first study that used the QTL approach to solve the genetic basis of tolerance to low pH in yeast. The knowledge provided here may help develop more robust strains for ethanol production and also other yeast-based industrial processes.

## Methods

### Strains and plasmids

A total of 41 *Saccharomyces sp*. strains (Additional file 4: Table S2) were used for the screening of low-pH tolerance phenotype in yeast. Diploid industrial *S. cerevisiae* PE-2 [19] was used as the acid-resistant reference strain, and ACY_503 (PE-2, MATa) its superior tolerant haploid. Non-tolerant laboratory MATα CEN.PK113-1A was used for the crossing with ACY_503 to generate hybrid ACY_503/CEN.PK113-1A, whose F_2_ progeny was used in low pH assays. The main *S. cerevisiae* strains used in this work are presented in Table 4. *Escherichia coli* DH5α was used in cloning procedures for plasmids used in this study. Plasmid pMF002 (pMATa-EGFP-tMATa; tMATα-CyOFP-pMATα; KanMX) was used for high-throughput separation and collection of segregants using flow cytometry.

**Table 4:** Main *Saccharomyces cerevisiae* strains used in this work

Strain	Description	Source
PE-2	Brazilian ethanol mill indigenous diploid strain	Basso et al. [5]
CEN.PK113-1A (CEN.PK)	*MATα* (prototrophic)	Euroscarf
ACY503	*MATa,* haploid segregant from PE-2	This study
ACY503/CEN.PK113-1A	Hybrid diploid strain from the crossing of ACY503 and CEN.PK113-1A	This study
ACY503 *gas1*/CEN.PK *gas1Δ*	ACY503 crossed with CEN.PK113-1A *gas1Δ*	This study
ACY503 *Δgas1*/CEN.PK *gas1*	ACY503 *gas1Δ* crossed with CEN.PK113-1A	This study
ACY503 *elp6*/CEN.PK *elp6Δ*	ACY503 crossed with CEN.PK113-1A *elp6Δ*	This study
ACY503 *Δelp6*/CEN.PK *elp6*	ACY503 *elp6Δ* crossed with CEN.PK113-1A	This study
ACY503 *fet4*/CEN.PK *fet4Δ*	ACY503 crossed with CEN.PK113-1A *fet4Δ*	This study
ACY503 *fet4Δ*/CEN.PK *fet41*	ACY503 *fet4Δ* crossed with CEN.PK113-1A	This study
ACY503 *glc8*/CEN.PK *glc8Δ*	ACY503 crossed with CEN.PK113-1A *glc8Δ*	This study
ACY503 *glc8Δ*/CEN.PK *glc8*	ACY503 *glc8Δ* crossed with CEN.PK113-1A	This study

### Growth media

YPD medium (10 g/L yeast extract; 20 g/L peptone; 20 g/L d-glucose), solidified with 15 g/L agar when required, was used for yeast propagation. G418 (200 µg/mL), hygromycin B (300 mg/ml) were added to the medium for the selection of strains with a KanMX or hphMX6 resistant marker, respectively. For stress-screening procedures, 1 M H_2_SO_4_ was used to adjust YPD pH before autoclaving. For sporulation procedures, 1% (m/v) KAc supplemented with a complete drop-out solution (460 mg/mL) was used. Cultivation occurred at 30 ºC and 250 rpm unless otherwise noticed. Bacteria were grown in Luria–Bertani (LB) broth (10 g/L tryptone, 10 g/L NaCl, 5 g/L yeast extract) supplemented with 100 μg/mL ampicillin at 37 ºC.

### General molecular biology

Yeast transformation was carried out with the PEG/LiAc method [78]. Transformation of the DH10β E. coli cells was done by the standard heat shock method. Bacterial plasmid purification was performed with a standard miniprep protocol [79]. Genomic DNA was extracted with (LiOAc)-SDS/EtOH fast protocol [80] for PCR purposes. All PCR reactions were performed with Phusion^®^ High Fidelity DNA Polymerase following the manufacturer’s instructions (NEB—New England Biolabs).

### F_1_ mating and sporulation

ACY503 and CEN.PK113-1A strains were matted by scratching them over an YPD plate. Ploidy of isolated colonies was checked by PCR and the diploid strain ACY503/CEN.PK113-1A was obtained. The diploid strain was further transformed with plasmid pMF_002. Sporulation was induced in conical glass tubes containing 2 mL of 1% KAc medium and 100 µL of a saturated culture of the diploid ACY503/CEN.PK113-1A expressing pMF_002. Tetrad formation was checked by microscope and when 80% of the culture has formed by tetrads, the cells were harvested and submitted to an asci lysis assay.

### F_2_ spore disruption and segregant collection

ACY_503/CEN.PK113-1A spore disruption was performed as follows: 250 µL of pelleted sporulated culture were resuspended in 100 µL of micromanipulation buffer (1 M sorbitol) with 1 µL of β-mercaptoethanol previously added. After, 16 µL of cells was added to a new Eppendorf tube containing 20 µL of Lyticase (0.5 mg/mL). The tube was vortexed and incubated at 30 °C during 3 h with shaking at 900 rpm. Digestion was checked by light microscopy and stopped with the addition of 100 µL of distilled water. The cell suspension was then vortexed for 2 min and centrifuged for 1 min, the supernatant was removed and the tube was sonicated twice for 1 min at level 2 (20%) and then diluted to OD_660_ = 0.4 in 1X PBS.

BD FACSAria flow cytometer III (BD-Bioscience) coupled with a cell sorter was used to sort 500,000 green (515/545) or orange (655/695) fluorescent cells each, using a FITC filter (488 nm). Sorted cells were added to 10 mL YPD containing 100 µg/mL ampicillin in glass tubes and grown for 6 h. Cultures were spun down for 5 min at 3,000 rpm and after supernatant removal cells were resuspended in 950 µl water. 200 µL of cell suspension were then plated in low-pH media ranging from 1.9 to 4. Plates were grown for 120 h and ploidy of the hypothetical segregants was assessed by multiplex PCR. In summary, genomic DNA from each colony was extracted using the protocol described by Looke [80]. Further, the extracted DNA was used as template for a multiplex PCR reaction using two distinct pairs of primers that anneal specifically to the MATa or MAT**α** locus. The presence of a single band at 500 or 600 bp indicates that the analyzed colony poses a MATa or MAT**α** locus, respectively. The presence of both bands in a single reaction indicates the presence of both loci in the same analyzed colony indicating that cells are diploids.

### Colony spot assay for *Saccharomyces sp.* screening at low-pH conditions

Yeast phenotyping was performed as described by Takeshi et al. [81]. Initially, strains were grown overnight in 96-well plates containing 200 μL YPD. Plates were then vigorously shaken to disperse cells and a replicator block tool was used to inoculate strains on solid plates containing different low-pH conditions. Replicates were performed in three randomized positions to minimize technical errors. Growth of each strain was assessed by colony size captured by plate image using Gel Doc™ XR + Gel Documentation System (BIO-RAD, USA). The dimensions of all the images were set at 13.4 × 10 cm (W × L) and imaged under white Epi illumination with 0.5 s exposure time. Every colony pixel intensity was measured using ImageJ. The total pixel intensity within a circle (spot radius = 50 pixels) surrounding each colony in the image was measured using the Plate Analysis JRU v1 plugin for ImageJ (http://research.stowers.org/imagejplugins/index.html). The average pixel intensity was determined by dividing the total pixel intensity by the area of the circle examined (7845 pixels2). Finally, relative growth is calculated as the ratio between the average pixel intensity of the strain colony growing on low-pH media and control (YPD) condition.

### Preparation of DNA samples

Parental strains ACY_503 and CEN.PK113-1A, as well as 79 superior and inferior segregants pools for low pH assay were individually inoculated in 2 mL YPD medium and grown to the stationary phase at 30 °C. The genomic DNA was pooled extracted using the methods described by Pais et al. [82]. For each pool and condition, OD_660_ of each culture was individually assessed and the cells were mixed in equivalent concentrations to form a heterogeneous pool containing approximately the same cell concentration representative for each strain. DNA was extracted according to the procedures described by Ausubel et al. [83] and DNA concentration and quality were estimated with a Nanodrop 3000 UV–Vis spectrophotometer (Wilmington, DE, USA). The two pools and parental DNA were prepared for Illumina sequencing.

### Pooled-segregant whole-genome sequence analysis and QTL mapping

At least 5 μg of gDNA from the superior and inferior low-pH resistance phenotype pools and parental strains were provided to Central Laboratory of High Performance Technologies in Life Sciences (LaCTAD) from the University of Campinas (UNICAMP) for whole-genome sequencing using the Illumina HiSeq 2500 platform. 2 × 100 paired-end reads were generated and aligned to the genome sequence of the CEN.PK113-7D reference strain [48]. Bowtie2 program version 2.3.5.1 [84] was used to align the paired-end reads of each sample against the reference genome. Alignment files were converted to BAM files using samtools software version 1.3.1 [85]. In addition, potential PCR duplicates were removed using picard version 2.23.9 command “MarkDuplicates” [86], in which if multiple read pairs have identical external coordinates, only the pair with the highest mapping quality is retained. SNP calling was performed using GATK (v4.0.12.0) base quality score recalibration, indel realignment and SNP and INDEL discovery and genotyping across the two samples (hard and low resistance pool) using standard high filtering parameters [66]. For each pool the ploidy level was configured to 79 (number of haploid individuals in each pool).

The statistical analysis of QTLs was performed as proposed by Magwene et al. [87] using QTLseqR package version 0.7.0. A modified G statistic is calculated for each SNP based on the observed and expected allele depths and the value is smoothed using a Nadaraya–Watson, or tricube smoothing kernel. This smoothing method weights neighboring SNPs’ G statistic by their relative distance from the focal SNP such that closer SNPs receive higher weights. The analysis was performed with an R package called QTLseqr [88]. In short, the command runGprimeAnalysis() was used to calculate the G statistic for each SNP. It then counts the number of SNPs within the set window bandwidth and estimates the tricube-smoothed G’ and ∆(SNP-index) values of each SNP within that window. For both analyses a sliding window of 80 kb size was used to calculate G’ of each SNP. The results were presented as – log(p-value) since p-values can be estimated from the null distribution of G’, which assumes no QTL and provides a more direct statistical interpretation of QTL mapping.

### Reciprocal hemizygosity analysis (RHA)

Hemizygous diploid strains were constructed by deleting the evaluated gene in one of the parental strains and crossing the haploid mutant with the opposite mating type parental strain. For example, to create the hemizygous diploid strain for *GAS1* allele, a knockout of the gene was performed on ACY_503 parental strain and further crossed to wild-type CEN.PK113-1A, creating the heterozygous diploid ACY_503/CEN.PK113-1A GAS1Δ/GAS1. The opposite strategy was made to obtain the heterozygous diploid strain ACY_503/CEN.PK113-1A GAS1/GAS1Δ. Genetic modifications were checked via PCR.

### Acid wash treatment assay

The CEN.PK113-1A and CEN.PK113-1A *GAS1*^ACY_503^ strains were cultivated overnight until reaching stationary phase (12–16 h). Further, cells were washed 3 × with water to remove media and diluted on a water solution with pH 1.5 corrected by addition of H_2_SO_4_ 3 M. Cells were kept on this solution under low agitation (50 rpm) and aliquots were taken every hour to assess cell viability. Cell viability was measured by staining an aliquot of cells (10 µL) with the same volume of Trypan Blue 0,4%. The solution was properly diluted and cells counted using a Neubauer chamber. The dye exclusion test is based upon the concept that viable cells do not take up impermeable dyes (like Trypan Blue), but dead cells are permeable and take up the dye.

## Supplementary Information

**Additional file 1.** Phenotypic distribution of 48 JAY270 segregants growing on solid YPD containing decreasing concentrations of pH to define the Minimum Inhibitory Concentration. A. Boxplot graph containing the colony size values of 48 JAY270 segregants growing on solid YPD at different pH values (4; 3.5; 3; 2.5; 2.1). B. Images of each low pH plate containing the 48 segregants evaluated on this minimum inhibitory concentration assay. The images were captured 96 hours after plating.

**Additional file 2**. Spotting assay for growing at low pH (2.1) of ACY503/CEN.PK113-1A segregants belonging to the “high resistant pool” (A) and “low resistant pool” (B). Each lane represents a different isolated colony and the columns the dilution factor applied to the initial cultures before spotting.

**Additional file 3**. GAS1 mutation profile of 1053 distinct *Saccharomyces cerevisiae* strains.

**Additional file 4**. List of 41 Saccharomyces sp. strains evaluated during growth at low pH (2.5).

## Declarations

### Availability of data and materials

The complete dataset of DNA-seq reads have been deposited in SRA under accession number PRJNA755995.
