# Supplementary figures and images for: QTL mapping of a Brazilian bioethanol strain links the cell wall protein-encoding gene GAS1 to low pH tolerance in S. cerevisiae
Source: Biotechnol Biofuels. 2021 Dec 16;14:239. doi: 10.1186/s13068-021-02079-6 (PMC8675505; doi:10.1186/s13068-021-02079-6)

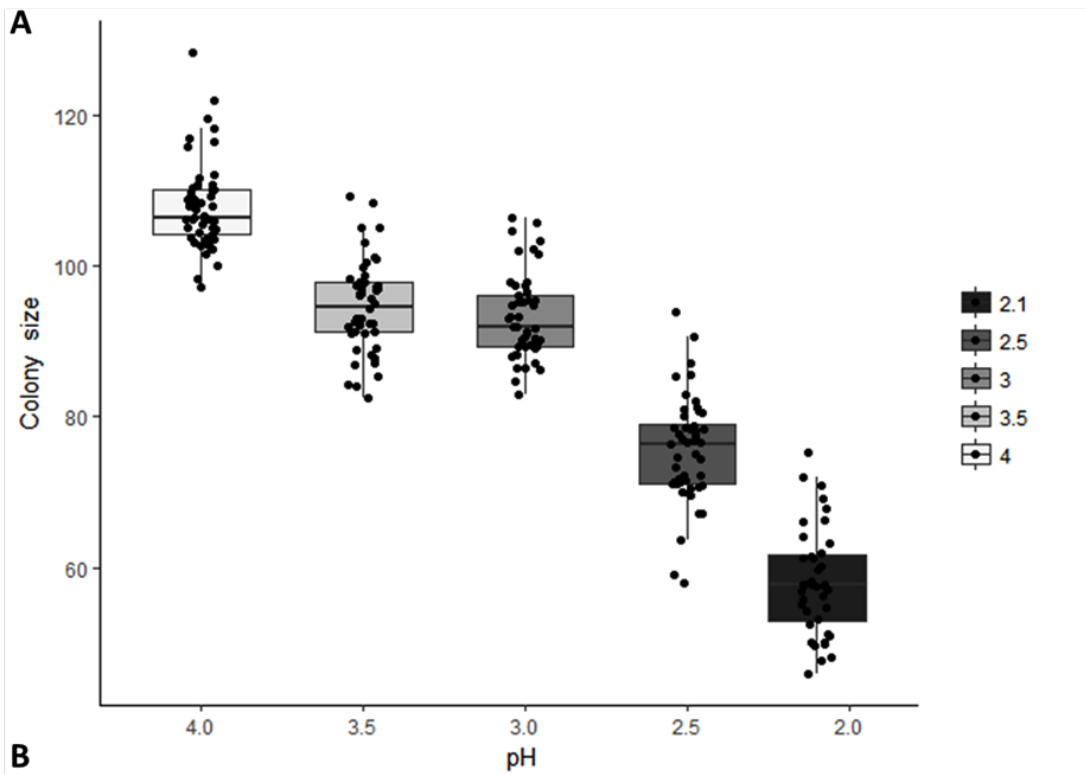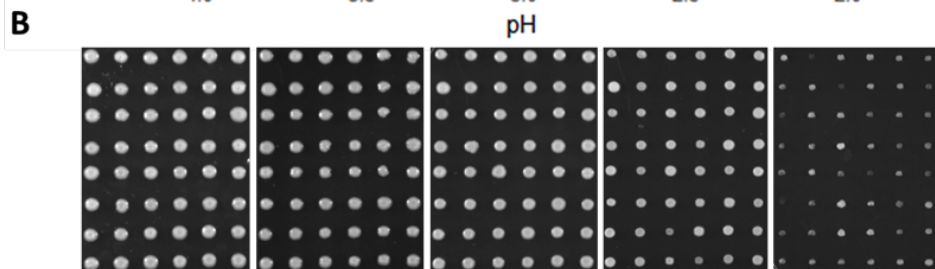

Supplement: Supplementary file 1 — Additional file 1. Phenotypic distribution of 48 JAY270 segregants growing on solid YPD containing decreasing concentrations of pH to define the Minimum Inhibitory Concentration. A. Boxplot graph containing the colony size values of 48 JAY270 segregants growing on solid YPD at different pH values (4; 3.5; 3; 2.5; 2.1). B. Images of each low pH plate containing the 48 segregants evaluated on this minimum inhibitory concentration assay. The images were captured 96 hours after plating. [file 13068_2021_2079_MOESM1_ESM.pdf]

**A**

# High tolerance pool

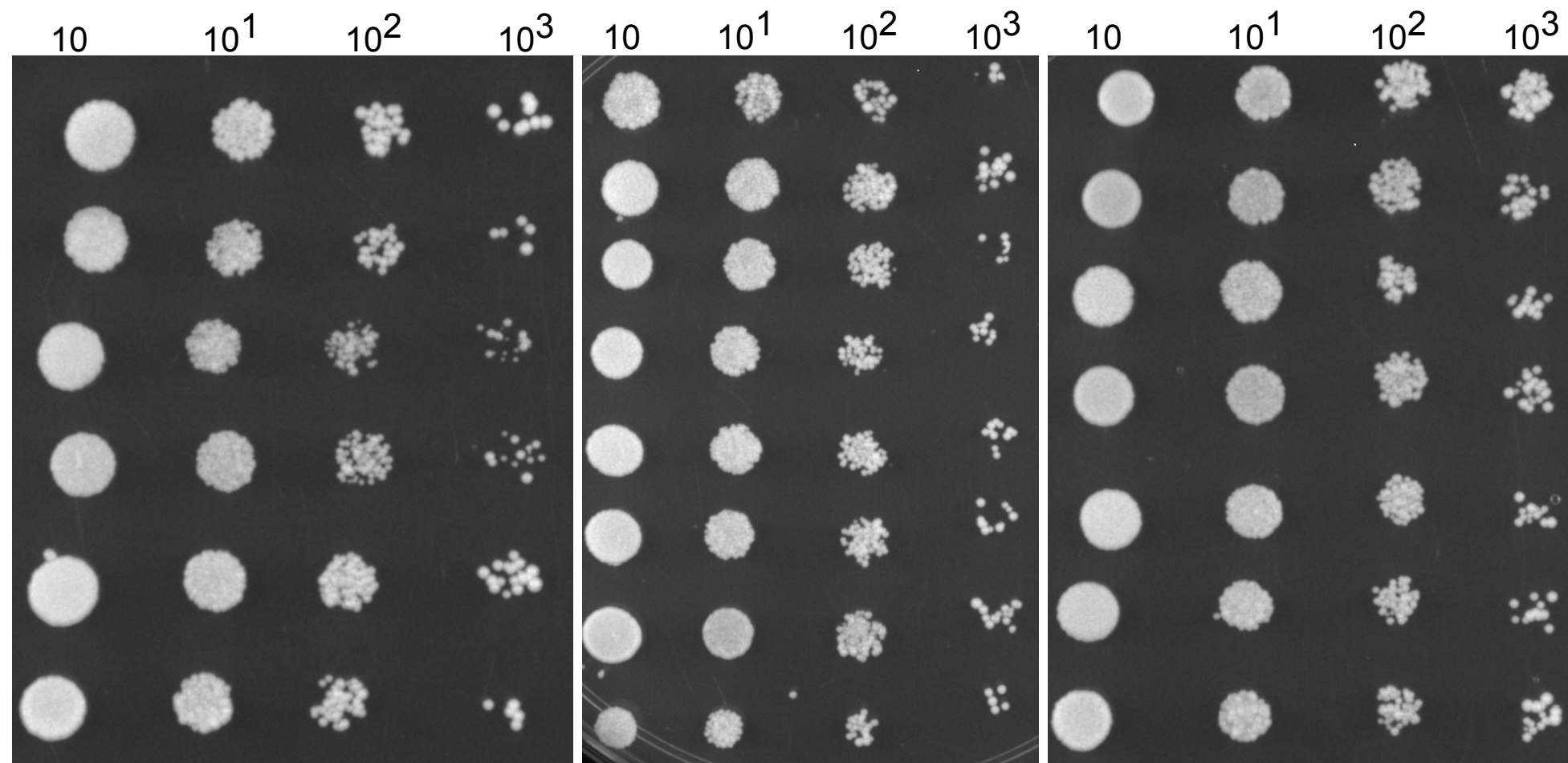**B**

# Low tolerance pool

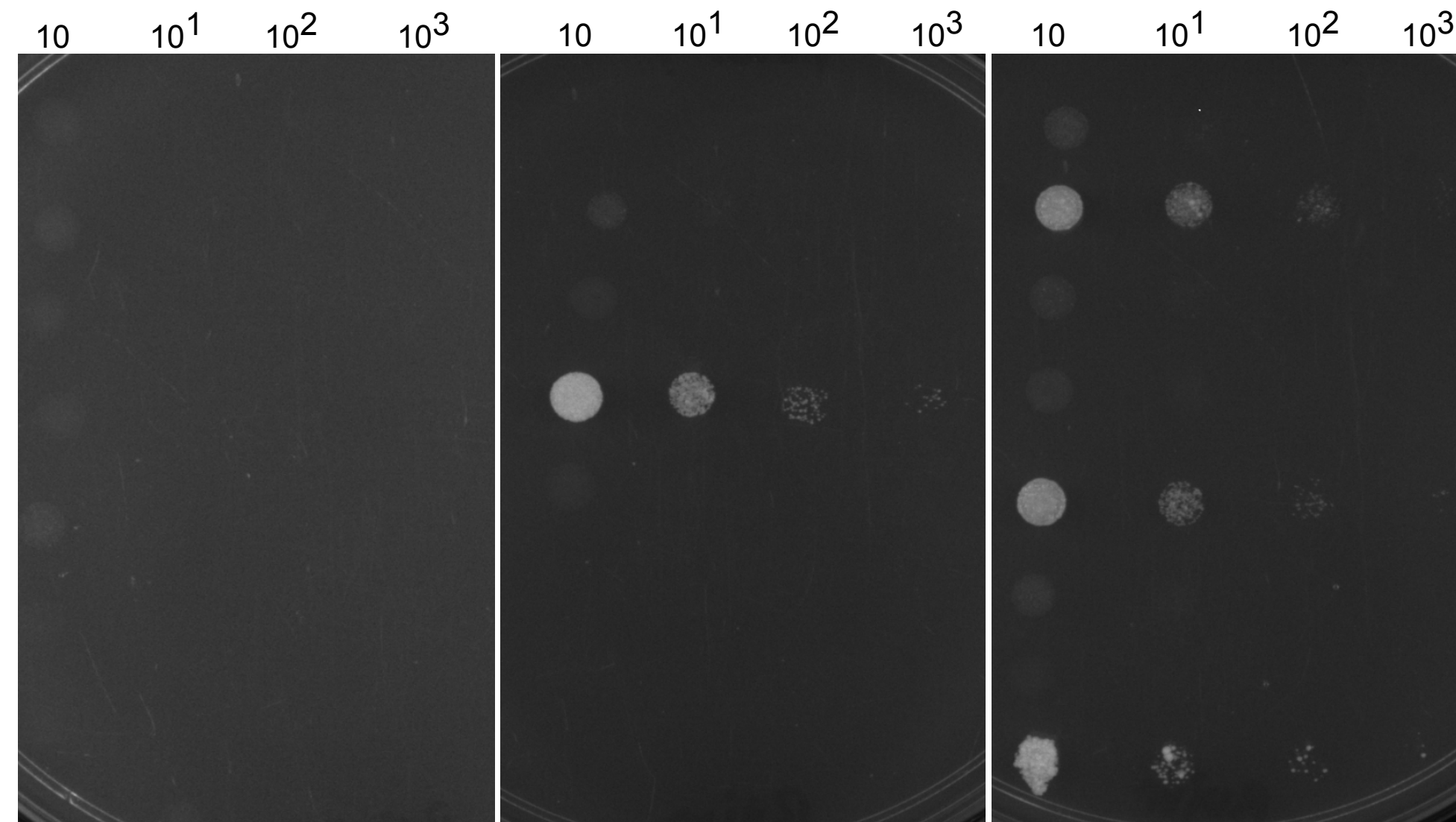

Supplement: Supplementary file 2 — Additional file 2. Spotting assay for growing at low pH (2.1) of ACY503/CEN.PK113-1A segregants belonging to the “high resistant pool” (A) and “low resistant pool” (B). Each lane represents a different isolated colony and the columns the dilution factor applied to the initial cultures before spotting. [file 13068_2021_2079_MOESM2_ESM.pdf]
