# Supplementary material for: QTL mapping of a Brazilian bioethanol strain links the cell wall protein-encoding gene GAS1 to low pH tolerance in S. cerevisiae
Source: Biotechnol Biofuels. 2021 Dec 16;14:239. doi: 10.1186/s13068-021-02079-6 (PMC8675505; doi:10.1186/s13068-021-02079-6)
Supplement: Supplementary file 4 — Additional file 4. List of 41 Saccharomyces sp. strains evaluated during growth at low pH (2.5). [file 13068_2021_2079_MOESM4_ESM.docx]

Supplementary table S2: List of 41 Saccharomyces sp. strains evaluated during growth at low pH (2.5).

| Identification | Specie | Origin |
| --- | --- | --- |
| IZ137 | *Saccharomyces cerevisiae* | Personal collection Dr. Luis Humberto Gomes ESALQ/USP |
| IZ0267 | *Saccharomyces cerevisiae* | Personal collection Dr. Luis Humberto Gomes ESALQ/USP |
| IZ0287 | *Saccharomyces cerevisiae var ellipsoideus* | Personal collection Dr. Luis Humberto Gomes ESALQ/USP |
| IZ0310 | *Saccharomyces cerevisiae* | Personal collection Dr. Luis Humberto Gomes ESALQ/USP |
| IZ0651 | *Saccharomyces cerevisiae var ellipsoideus* | Personal collection Dr. Luis Humberto Gomes ESALQ/USP |
| IZ0658 | *Saccharomyces cerevisiae* | Personal collection Dr. Luis Humberto Gomes ESALQ/USP |
| IZ0659 | *Saccharomyces cerevisiae* | Personal collection Dr. Luis Humberto Gomes ESALQ/USP |
| IZ0662 | *Saccharomyces cerevisiae* | Personal collection Dr. Luis Humberto Gomes ESALQ/USP |
| IZ0671 | *Saccharomyces cerevisiae* | Personal collection Dr. Luis Humberto Gomes ESALQ/USP |
| IZ0672 | *Saccharomyces cerevisiae* | Personal collection Dr. Luis Humberto Gomes ESALQ/USP |
| IZ0677 | *Saccharomyces cerevisiae* | Personal collection Dr. Luis Humberto Gomes ESALQ/USP |
| IZ1169 | *Saccharomyces cerevisiae* | Personal collection Dr. Luis Humberto Gomes ESALQ/USP |
| IZ1215 | *Saccharomyces cerevisiae* | Personal collection Dr. Luis Humberto Gomes ESALQ/USP |
| IZ1348 | *Saccharomyces cerevisiae* | Personal collection Dr. Luis Humberto Gomes ESALQ/USP |
| IZ1349 | *Saccharomyces cerevisiae* | Personal collection Dr. Luis Humberto Gomes ESALQ/USP |
| IZ1350 | *Saccharomyces cerevisiae* | Personal collection Dr. Luis Humberto Gomes ESALQ/USP |
| IZ1716 | *Saccharomyces cerevisisae* | Personal collection Dr. Luis Humberto Gomes ESALQ/USP |
| IZ1832 | *Saccharomyces cerevisiae* | Personal collection Dr. Luis Humberto Gomes ESALQ/USP |
| IZ2003 | *Saccharomyces cerevisiae* | Personal collection Dr. Luis Humberto Gomes ESALQ/USP |
| IZ2004 | *Saccharomyces cerevisiae* | Personal collection Dr. Luis Humberto Gomes ESALQ/USP |
| ATCC04132 | *Saccharomyces cerevisiae* | American Type Culture Collection - 1982 |
| ATCC24858 | *Saccharomyces cerevisiae* | American Type Culture Collection - 1982 |
| ATCC26603 | *Saccharomyces cerevisiae* | American Type Culture Collection - 1982 |
| FT116L | *Saccharomyces cerevisiae* | Personal collection Dr. Luis Humberto Gomes ESALQ/USP |
| FT119L | *Saccharomyces cerevisiae* | Personal collection Dr. Luis Humberto Gomes ESALQ/USP |
| M4-10A | *Saccharomyces cerevisiae* | Personal collection Dr. Luis Humberto Gomes ESALQ/USP |
| M4-10B | *Saccharomyces cerevisiae* | Personal collection Dr. Luis Humberto Gomes ESALQ/USP |
| PE-2 | *Saccharomyces cerevisiae* | Personal collection Dr. Luis Humberto Gomes ESALQ/USP |
| BG-1 | *Saccharomyces cerevisiae* | Personal collection Dr. Luis Humberto Gomes ESALQ/USP |
| Y01347 | *Saccharomyces cerevisiae* | Personal collection Dr. Luis Humberto Gomes ESALQ/USP |
| Y14620 | *Saccharomyces cerevisiae* | EUROSCARF |
| Rad317-8C | *Saccharomyces cerevisiae* | Personal collection Dr. Luis Humberto Gomes ESALQ/USP |
| ANTARTICA 5 | *Saccharomyces cerevisiae* | Personal collection Dr. Luis Humberto Gomes ESALQ/USP |
| ATCC28097 | *Saccharomyces uvarum* | American Type Culture Collection - 1982 |
| ATCC28099 | *Saccharomyces uvarum* | American Type Culture Collection - 1982 |
| ATCC28100 | *Saccharomyces uvarum* | American Type Culture Collection - 1982 |
| FLRT2 | *Saccharomyces boulardii* | Personal collection Dr. Luis Humberto Gomes ESALQ/USP |
| CAT-1 | *Saccharomyces cerevisiae* | Personal collection Dr. Luis Humberto Gomes ESALQ/USP |
| SA-1 | *Saccharomyces cerevisiae* | Personal collection Dr. Luis Humberto Gomes ESALQ/USP |
| J132b | *Saccharomyces cerevisiae var diastaticus* | Personal collection Dr. Luis Humberto Gomes ESALQ/USP |
